# Supplementary material for: Genetic and phenotypic differentiation of lumpfish (Cyclopterus lumpus) across the North Atlantic: implications for conservation and aquaculture
Source: PeerJ. 2018 Nov 20;6:e5974. doi: 10.7717/peerj.5974 (PMC6251346; doi:10.7717/peerj.5974)
Supplement: Table S1 [file peerj-06-5974-s002.docx]

**Table S1**. Details of 10 microsatellite loci (Skirnisdottir et al. 2013) used in this study, including allele sizes found across study populations.

| Locus | Primer sequence (5' - 3') | Dye | Allele size range (bp) |
| --- | --- | --- | --- |
| Multiplex 1 |  |  |  |
| *Clu29* | F: CGCGCGGTCAGCTCATCCTTAG | PET | 126-142 |
|  | R: TCGCGTGACGGACAGGTTTCG |  |  |
| *Clu34* | F: TCTGCGATAGTAGCGTCAGGGTTC | NED | 179-219 |
|  | R: AGGCCGGCTGATCAAGAGCAC |  |  |
| *Clu36* | F: CACGGCGAGTCAGACGAGGC | 6-FAM | 183-203 |
|  | R: GCTGCCGCTACTCCGCACAG |  |  |
| *Clu45* | F: GCGCAGGAATGCGCCTGAAG | PET | 268-298 |
|  | R: ACCGCAGCTTGTTGGGCAGG |  |  |
|  |  |  |  |
| Multiplex 2 |  |  |  |
| *Clu12* | F: CCACAACCGGTGGGTCCCG | 6-FAM | 188-202 |
|  | R: ACGCTCCTTCTGATCTTCGCCC |  |  |
| *Clu26* | F: CGAGAGAGGAGAACGCACGGC | 6-FAM | 97–121 |
|  | R: GGCACAAGTGCATGGGCACG |  |  |
| *Clu33* | F: TCATGCAAGCATTTGAGCGCCG | VIC | 173–193 |
|  | R: TGTTGCCTTGTAACTGCGCTTGAG |  |  |
| *Clu37* | F: CTTCACAGGTCGGGCGACGG | PET | 200-226 |
|  | R: GCACAGCGATGACGCTTGCAG |  |  |
| *Clu40* | F: TGGGCATACAGGTCTGAACACGC | NED | 246-266 |
|  | R: GCCACCTGCTGCAGCCTCTC |  |  |
| *Clu44* | F: CCGGCCCAGCCTGCCTTATG | 6-FAM | 269-287 |
|  | R: TGCCTGGAAACAGTGTATGGCAC |  |  |
