# Supplementary material for: Genetic and phenotypic differentiation of lumpfish (Cyclopterus lumpus) across the North Atlantic: implications for conservation and aquaculture
Source: PeerJ. 2018 Nov 20;6:e5974. doi: 10.7717/peerj.5974 (PMC6251346; doi:10.7717/peerj.5974)
Supplement: Table S2 [file peerj-06-5974-s003.docx]

**Table S2.** Presence of null alleles identified at 10 microsatellite loci across 15 lumpfish populations using Microchecker v.2.2.3 (Van Oosterhout et al. 2004; *0.05, ** 0.01, ***0.001).

| Popn | *Clu*  *29* | *Clu*  *34* | *Clu*  *36* | *Clu*  *45* | *Clu*  *12* | *Clu*  *26* | *Clu*  *33* | *Clu*  *37* | *Clu*  *40* | *Clu44* |
| --- | --- | --- | --- | --- | --- | --- | --- | --- | --- | --- |
| FB |  |  |  |  | 0.175* |  |  |  |  |  |
| CB |  |  |  |  |  |  | 0.189** |  |  |  |
| WB |  |  |  |  | 0.140* |  |  |  | 0.143*** |  |
| Ha |  |  |  |  |  | 0.310*** |  |  | 0.213*** |  |
| Kl |  | 0.193** |  |  |  |  |  |  |  |  |
| VB |  |  |  |  |  | 0.145* |  |  |  |  |
| OH |  |  |  |  |  |  |  |  |  |  |
| We |  |  |  |  |  |  |  |  |  |  |
| Gu |  | 0.289*** |  |  |  |  |  |  |  |  |
| Na |  | 0.181* |  |  |  | 0.168* |  | 0.243*** |  |  |
| Av |  |  |  |  |  |  |  |  |  |  |
| Ro |  | 0.387*** |  |  |  |  |  |  |  |  |
| KB |  |  |  |  |  |  |  |  |  |  |
| Öl |  |  | 0.209*** |  |  |  | 0.242** |  |  |  |
| GS |  | 0.282*** | 0.197*** |  | 0.204** |  | 0.185* |  | 0.121* |  |
