# Supplementary material for: Genetic and phenotypic differentiation of lumpfish (Cyclopterus lumpus) across the North Atlantic: implications for conservation and aquaculture
Source: PeerJ. 2018 Nov 20;6:e5974. doi: 10.7717/peerj.5974 (PMC6251346; doi:10.7717/peerj.5974)
Supplement: Table S3 [file peerj-06-5974-s004.docx]

**Table S3**. Pairwise *F_ST_* values of 9 microsatellite loci (*Clu12* removed) across 15 populations, * denotes significant value after Bonferroni correction (*P* < 0.00022).

|  | FB | CB | WB | Ha | Kl | VB | OH | We | Gu | Na | Av | Ro | KB | Öl | GS |
| --- | --- | --- | --- | --- | --- | --- | --- | --- | --- | --- | --- | --- | --- | --- | --- |
| FB |  | *NS* | * | * | * | * | * | * | * | * | * | * | * | * | * |
| CB | 0.011 |  | * | * | * | * | * | * | * | * | * | * | * | * | * |
| WB | 0.027 | 0.021 |  | * | * | * | * | * | * | * | * | * | * | * | * |
| Ha | 0.146 | 0.122 | 0.129 |  | * | * | * | * | * | * | * | * | * | * | * |
| Kl | 0.131 | 0.113 | 0.115 | 0.060 |  | *NS* | * | * | * | * | * | * | * | * | * |
| VB | 0.126 | 0.106 | 0.113 | 0.046 | 0.012 |  | * | * | * | * | * | * | *NS* | * | * |
| OH | 0.154 | 0.118 | 0.131 | 0.047 | 0.034 | 0.017 |  | * | * | *NS* | * | * | * | * | * |
| We | 0.148 | 0.136 | 0.124 | 0.056 | 0.041 | 0.030 | 0.018 |  | *NS* | * | * | * | * | * | * |
| Gu | 0.163 | 0.146 | 0.139 | 0.081 | 0.051 | 0.047 | 0.016 | 0.000 |  | * | * | * | * | * | * |
| Na | 0.150 | 0.121 | 0.128 | 0.069 | 0.029 | 0.015 | 0.000 | 0.022 | 0.023 |  | * | * | * | * | * |
| Av | 0.130 | 0.113 | 0.099 | 0.114 | 0.035 | 0.029 | 0.050 | 0.053 | 0.052 | 0.027 |  | * | * | * | * |
| Ro | 0.151 | 0.138 | 0.150 | 0.058 | 0.039 | 0.028 | 0.031 | 0.039 | 0.045 | 0.025 | 0.074 |  | * | * | * |
| KB | 0.120 | 0.097 | 0.107 | 0.037 | 0.022 | 0.007 | 0.024 | 0.039 | 0.059 | 0.016 | 0.044 | 0.034 |  | * | * |
| Ö | 0.202 | 0.167 | 0.193 | 0.131 | 0.124 | 0.150 | 0.139 | 0.143 | 0.149 | 0.156 | 0.176 | 0.172 | 0.116 |  | *NS* |
| GS | 0.209 | 0.186 | 0.207 | 0.136 | 0.134 | 0.159 | 0.161 | 0.157 | 0.172 | 0.175 | 0.195 | 0.171 | 0.129 | 0.000 |  |
