# Supplementary material for: Genetic and phenotypic differentiation of lumpfish (Cyclopterus lumpus) across the North Atlantic: implications for conservation and aquaculture
Source: PeerJ. 2018 Nov 20;6:e5974. doi: 10.7717/peerj.5974 (PMC6251346; doi:10.7717/peerj.5974)
Supplement: Table S4 [file peerj-06-5974-s005.docx]

**Table S4**. Pairwise *F_ST_* values of 9 microsatellite loci (*Clu33* removed) across 15 populations, * denotes significant value after Bonferroni correction (*P* < 0.00022).

|  | FB | CB | WB | Ha | Kl | VB | OH | We | Gu | Na | Av | Ro | KB | Öl | GS |
| --- | --- | --- | --- | --- | --- | --- | --- | --- | --- | --- | --- | --- | --- | --- | --- |
| FB |  | *NS* | * | * | * | * | * | * | * | * | * | * | * | * | * |
| CB | 0.011 |  | * | * | * | * | * | * | * | * | * | * | * | * | * |
| WB | 0.017 | 0.023 |  | * | * | * | * | * | * | * | * | * | * | * | * |
| Ha | 0.159 | 0.124 | 0.129 |  | * | * | * | * | * | * | * | * | * | * | * |
| Kl | 0.125 | 0.107 | 0.115 | 0.039 |  | *NS* | * | * | * | * | * | * | *NS* | * | * |
| VB | 0.131 | 0.104 | 0.121 | 0.037 | 0.005 |  | * | * | * | *NS* | *NS* | * | *NS* | * | * |
| OH | 0.171 | 0.128 | 0.151 | 0.038 | 0.033 | 0.018 |  | * | *NS* | *NS* | * | * | * | * | * |
| We | 0.181 | 0.156 | 0.156 | 0.058 | 0.048 | 0.032 | 0.023 |  | *NS* | * | * | * | * | * | * |
| Gu | 0.182 | 0.158 | 0.162 | 0.071 | 0.054 | 0.045 | 0.016 | 0.000 |  | * | * | * | * | * | * |
| Na | 0.160 | 0.128 | 0.152 | 0.050 | 0.026 | 0.011 | 0.000 | 0.013 | 0.018 |  | * | * | * | * | * |
| Av | 0.106 | 0.094 | 0.098 | 0.075 | 0.026 | 0.014 | 0.043 | 0.039 | 0.046 | 0.026 |  | * | * | * | * |
| Ro | 0.173 | 0.154 | 0.168 | 0.056 | 0.027 | 0.031 | 0.033 | 0.055 | 0.048 | 0.027 | 0.057 |  | * | * | * |
| KB | 0.132 | 0.103 | 0.124 | 0.028 | 0.011 | 0.003 | 0.022 | 0.037 | 0.052 | 0.014 | 0.028 | 0.041 |  | * | * |
| Öl | 0.234 | 0.184 | 0.214 | 0.142 | 0.119 | 0.152 | 0.141 | 0.147 | 0.145 | 0.148 | 0.154 | 0.191 | 0.116 |  | *NS* |
| GS | 0.220 | 0.183 | 0.202 | 0.135 | 0.105 | 0.141 | 0.145 | 0.149 | 0.153 | 0.145 | 0.147 | 0.170 | 0.111 | 0.000 |  |
