# Supplementary material for: Genetic and phenotypic differentiation of lumpfish (Cyclopterus lumpus) across the North Atlantic: implications for conservation and aquaculture
Source: PeerJ. 2018 Nov 20;6:e5974. doi: 10.7717/peerj.5974 (PMC6251346; doi:10.7717/peerj.5974)
Supplement: Table S5 [file peerj-06-5974-s006.docx]

**Table S5**. Pairwise *F_ST_* values of 9 microsatellite loci (*Clu34* removed) across 15 populations, * denotes significant value after Bonferroni correction (*P* < 0.00022).

|  | FB | CB | WB | Ha | Kl | VB | OH | We | Gu | Na | Av | Ro | KB | Öl | GS |
| --- | --- | --- | --- | --- | --- | --- | --- | --- | --- | --- | --- | --- | --- | --- | --- |
| FB |  | *NS* | * | * | * | * | * | * | * | * | * | * | * | * | * |
| CB | 0.014 |  | * | * | * | * | * | * | * | * | * | * | * | * | * |
| WB | 0.024 | 0.018 |  | * | * | * | * | * | * | * | * | * | * | * | * |
| Ha | 0.161 | 0.118 | 0.133 |  | * | * | * | * | * | * | * | * | * | * | * |
| Kl | 0.139 | 0.106 | 0.110 | 0.062 |  | *NS* | * | * | * | * | * | * | * | * | * |
| VB | 0.141 | 0.100 | 0.113 | 0.049 | 0.014 |  | * | * | * | *NS* | * | * | *NS* | * | * |
| OH | 0.165 | 0.105 | 0.122 | 0.040 | 0.025 | 0.012 |  | * | * | *NS* | * | * | * | * | * |
| We | 0.171 | 0.132 | 0.126 | 0.056 | 0.043 | 0.030 | 0.022 |  | *NS* | * | * | * | * | * | * |
| Gu | 0.171 | 0.130 | 0.125 | 0.077 | 0.044 | 0.042 | 0.015 | 0.000 |  | * | * | * | * | * | * |
| Na | 0.161 | 0.107 | 0.118 | 0.060 | 0.018 | 0.006 | 0.000 | 0.023 | 0.022 |  | *NS* | * | *NS* | * | * |
| Av | 0.141 | 0.104 | 0.101 | 0.117 | 0.034 | 0.029 | 0.049 | 0.055 | 0.047 | 0.022 |  | * | * | * | * |
| Ro | 0.160 | 0.129 | 0.141 | 0.057 | 0.041 | 0.034 | 0.023 | 0.045 | 0.048 | 0.018 | 0.076 |  | * | * | * |
| KB | 0.143 | 0.098 | 0.118 | 0.038 | 0.025 | 0.007 | 0.017 | 0.036 | 0.050 | 0.005 | 0.047 | 0.034 |  | * | * |
| Öl | 0.237 | 0.180 | 0.211 | 0.136 | 0.135 | 0.153 | 0.132 | 0.136 | 0.137 | 0.146 | 0.180 | 0.180 | 0.118 |  | *NS* |
| GS | 0.231 | 0.190 | 0.216 | 0.138 | 0.139 | 0.158 | 0.149 | 0.148 | 0.157 | 0.160 | 0.196 | 0.174 | 0.129 | 0.000 |  |
