# Supplementary material for: Genetic and phenotypic differentiation of lumpfish (Cyclopterus lumpus) across the North Atlantic: implications for conservation and aquaculture
Source: PeerJ. 2018 Nov 20;6:e5974. doi: 10.7717/peerj.5974 (PMC6251346; doi:10.7717/peerj.5974)
Supplement: Table S6 [file peerj-06-5974-s007.docx]

**Table S6**. Pairwise *F_ST_* values of 9 microsatellite loci (*Clu36* removed) across 15 populations, * denotes significant value after Bonferroni correction (*P* < 0.00022).

|  | FB | CB | WB | Ha | Kl | VB | OH | We | Gu | Na | Av | Ro | KB | Öl | GS |
| --- | --- | --- | --- | --- | --- | --- | --- | --- | --- | --- | --- | --- | --- | --- | --- |
| FB |  | *NS* | * | * | * | * | * | * | * | * | * | * | * | * | * |
| CB | 0.012 |  | * | * | * | * | * | * | * | * | * | * | * | * | * |
| WB | 0.028 | 0.022 |  | * | * | * | * | * | * | * | * | * | * | * | * |
| Ha | 0.152 | 0.122 | 0.133 |  | * | * | * | * | * | * | * | * | * | * | * |
| Kl | 0.142 | 0.116 | 0.123 | 0.053 |  | *NS* | * | * | * | * | * | * | * | * | * |
| VB | 0.145 | 0.111 | 0.128 | 0.035 | 0.011 |  | * | * | * | * | * | * | *NS* | * | * |
| OH | 0.179 | 0.131 | 0.151 | 0.047 | 0.035 | 0.016 |  | * | * | *NS* | * | * | * | * | * |
| We | 0.187 | 0.156 | 0.159 | 0.060 | 0.054 | 0.034 | 0.023 |  | *NS* | * | * | * | * | * | * |
| Gu | 0.192 | 0.161 | 0.164 | 0.083 | 0.056 | 0.047 | 0.017 | 0.002 |  | * | * | * | * | * | * |
| Na | 0.180 | 0.137 | 0.154 | 0.068 | 0.036 | 0.015 | 0.000 | 0.025 | 0.025 |  | * | * | * | * | * |
| Av | 0.143 | 0.113 | 0.112 | 0.104 | 0.036 | 0.030 | 0.051 | 0.059 | 0.053 | 0.029 |  | * | * | * | * |
| Ro | 0.176 | 0.154 | 0.170 | 0.051 | 0.044 | 0.036 | 0.036 | 0.060 | 0.058 | 0.039 | 0.084 |  | * | * | * |
| KB | 0.142 | 0.107 | 0.125 | 0.035 | 0.023 | 0.002 | 0.026 | 0.044 | 0.063 | 0.017 | 0.041 | 0.043 |  | * | * |
| Öl | 0.230 | 0.184 | 0.216 | 0.147 | 0.134 | 0.148 | 0.147 | 0.149 | 0.154 | 0.163 | 0.176 | 0.191 | 0.121 |  | *NS* |
| GS | 0.218 | 0.189 | 0.213 | 0.142 | 0.133 | 0.152 | 0.163 | 0.158 | 0.169 | 0.177 | 0.189 | 0.176 | 0.126 | 0.000 |  |
