# Supplementary material for: Genetic and phenotypic differentiation of lumpfish (Cyclopterus lumpus) across the North Atlantic: implications for conservation and aquaculture
Source: PeerJ. 2018 Nov 20;6:e5974. doi: 10.7717/peerj.5974 (PMC6251346; doi:10.7717/peerj.5974)
Supplement: Table S7 [file peerj-06-5974-s008.docx]

**Table S7**. Pairwise *F_ST_* values of 9 microsatellite loci (*Clu37* removed) across 15 populations, * denotes significant value after Bonferroni correction (*P* < 0.00022).

|  | FB | CB | WB | Ha | Kl | VB | OH | We | Gu | Na | Av | Ro | KB | Öl | GS |
| --- | --- | --- | --- | --- | --- | --- | --- | --- | --- | --- | --- | --- | --- | --- | --- |
| FB |  | *NS* | * | * | * | * | * | * | * | * | * | * | * | * | * |
| CB | 0.012 |  | * | * | * | * | * | * | * | * | * | * | * | * | * |
| WB | 0.025 | 0.024 |  | * | * | * | * | * | * | * | * | * | * | * | * |
| Ha | 0.139 | 0.112 | 0.134 |  | * | * | * | * | * | * | * | * | * | * | * |
| Kl | 0.112 | 0.092 | 0.109 | 0.061 |  | *NS* | * | * | * | * | * | * | * | * | * |
| VB | 0.118 | 0.092 | 0.117 | 0.050 | 0.014 |  | * | * | * | * | * | * | *NS* | * | * |
| OH | 0.147 | 0.105 | 0.137 | 0.050 | 0.036 | 0.017 |  | * | * | *NS* | * | * | * | * | * |
| We | 0.148 | 0.126 | 0.139 | 0.065 | 0.052 | 0.034 | 0.021 |  | *NS* | * | * | * | * | * | * |
| Gu | 0.152 | 0.128 | 0.142 | 0.085 | 0.055 | 0.047 | 0.015 | 0.001 |  | * | * | * | * | * | * |
| Na | 0.151 | 0.114 | 0.142 | 0.075 | 0.035 | 0.017 | 0.000 | 0.023 | 0.024 |  | * | * | * | * | * |
| Av | 0.109 | 0.088 | 0.094 | 0.116 | 0.036 | 0.029 | 0.052 | 0.058 | 0.054 | 0.030 |  | * | * | * | * |
| Ro | 0.148 | 0.133 | 0.158 | 0.060 | 0.043 | 0.039 | 0.035 | 0.056 | 0.054 | 0.039 | 0.085 |  | * | * | * |
| KB | 0.115 | 0.087 | 0.115 | 0.040 | 0.024 | 0.006 | 0.025 | 0.041 | 0.058 | 0.017 | 0.045 | 0.043 |  | * | * |
| Öl | 0.224 | 0.180 | 0.221 | 0.145 | 0.135 | 0.157 | 0.147 | 0.146 | 0.151 | 0.164 | 0.183 | 0.192 | 0.125 |  | *NS* |
| GS | 0.212 | 0.184 | 0.217 | 0.141 | 0.135 | 0.159 | 0.164 | 0.159 | 0.170 | 0.178 | 0.194 | 0.179 | 0.133 | 0.000 |  |
