# Supplementary material for: Genetic and phenotypic differentiation of lumpfish (Cyclopterus lumpus) across the North Atlantic: implications for conservation and aquaculture
Source: PeerJ. 2018 Nov 20;6:e5974. doi: 10.7717/peerj.5974 (PMC6251346; doi:10.7717/peerj.5974)
Supplement: Table S8 [file peerj-06-5974-s009.docx]

**Table S8.** Pairwise *F_ST_* values of 9 microsatellite loci (*Clu40* removed) across 15 populations, * denotes significant value after Bonferroni correction (*P* < 0.00022).

|  | FB | CB | WB | Ha | Kl | VB | OH | We | Gu | Na | Av | Ro | KB | Öl | GS |
| --- | --- | --- | --- | --- | --- | --- | --- | --- | --- | --- | --- | --- | --- | --- | --- |
| FB |  | *NS* | * | * | * | * | * | * | * | * | * | * | * | * | * |
| CB | 0.010 |  | * | * | * | * | * | * | * | * | * | * | * | * | * |
| WB | 0.032 | 0.037 |  | * | * | * | * | * | * | * | * | * | * | * | * |
| Ha | 0.132 | 0.116 | 0.122 |  | * | * | * | * | * | * | * | * | * | * | * |
| Kl | 0.130 | 0.103 | 0.103 | 0.050 |  | * | * | * | * | * | * | * | * | * | * |
| VB | 0.129 | 0.100 | 0.112 | 0.046 | 0.013 |  | * | * | * | * | * | * | *NS* | * | * |
| OH | 0.165 | 0.124 | 0.139 | 0.053 | 0.035 | 0.023 |  | * | *NS* | *NS* | * | * | * | * | * |
| We | 0.195 | 0.166 | 0.160 | 0.058 | 0.060 | 0.044 | 0.021 |  | *NS* | * | * | * | * | * | * |
| Gu | 0.206 | 0.173 | 0.167 | 0.077 | 0.058 | 0.057 | 0.009 | 0.003 |  | * | * | * | * | * | * |
| Na | 0.177 | 0.133 | 0.144 | 0.080 | 0.041 | 0.023 | 0.001 | 0.033 | 0.027 |  | * | * | * | * | * |
| Av | 0.170 | 0.130 | 0.118 | 0.102 | 0.029 | 0.020 | 0.040 | 0.071 | 0.065 | 0.023 |  | * | * | * | * |
| Ro | 0.152 | 0.128 | 0.140 | 0.044 | 0.042 | 0.041 | 0.031 | 0.070 | 0.055 | 0.052 | 0.082 |  | * | * | * |
| KB | 0.125 | 0.090 | 0.104 | 0.034 | 0.026 | 0.007 | 0.031 | 0.054 | 0.072 | 0.026 | 0.041 | 0.048 |  | * | * |
| Öl | 0.198 | 0.148 | 0.190 | 0.077 | 0.123 | 0.112 | 0.105 | 0.107 | 0.127 | 0.125 | 0.155 | 0.129 | 0.083 |  | *NS* |
| GS | 0.208 | 0.173 | 0.205 | 0.102 | 0.155 | 0.151 | 0.157 | 0.158 | 0.178 | 0.185 | 0.198 | 0.163 | 0.127 | 0.005 |  |
