# Supplementary material for: Genetic and phenotypic differentiation of lumpfish (Cyclopterus lumpus) across the North Atlantic: implications for conservation and aquaculture
Source: PeerJ. 2018 Nov 20;6:e5974. doi: 10.7717/peerj.5974 (PMC6251346; doi:10.7717/peerj.5974)
Supplement: Table S9 [file peerj-06-5974-s010.docx]

**Table S9.** Global tests of deviation from Hardy-Weinberg equilibrium by heterozygote excess and heterozygote deficiency conducted in GenePop, across 15 populations of lumpfish genotyped using 10 microsatellite loci (*denotes significant deviation after Bonferroni correction).

| Population | Het excess | | Het deficiency | |
| --- | --- | --- | --- | --- |
|  | *P* | S.E. | *P* | S.E. |
| FB | 0.971 | 0.005 | 0.042 | 0.005 |
| CB | 0.955 | 0.005 | 0.060 | 0.007 |
| WB | 0.998 | 0.001 | 0.001* | 0.001 |
| Ha | 0.676 | 0.014 | 0.332 | 0.016 |
| Kl | 1.000 | 0.001 | 0.001* | 0.001 |
| VB | 0.904 | 0.011 | 0.099 | 0.008 |
| OH | 0.906 | 0.011 | 0.100 | 0.010 |
| We | 0.849 | 0.011 | 0.160 | 0.013 |
| Gu | 0.521 | 0.017 | 0.493 | 0.016 |
| Na | 0.994 | 0.002 | 0.004 | 0.001 |
| Av | 0.306 | 0.014 | 0.689 | 0.014 |
| Ro | 0.376 | 0.011 | 0.580 | 0.009 |
| KB | 0.996 | 0.001 | 0.006* | 0.001 |
| Öl | 1.000 | 0.001 | 0.001* | 0.001 |
| GS | 1.000 | 0.001 | 0.001* | 0.001 |
