# Supplementary material for: Genetic and phenotypic differentiation of lumpfish (Cyclopterus lumpus) across the North Atlantic: implications for conservation and aquaculture
Source: PeerJ. 2018 Nov 20;6:e5974. doi: 10.7717/peerj.5974 (PMC6251346; doi:10.7717/peerj.5974)
Supplement: Table S10 [file peerj-06-5974-s011.docx]

**Table S10.** Results of AMOVA detailing degrees of freedom (df), sum of squares (SS), mean of squares (MS) and estimated variance of each source for 15 populations of lumpfish genotyped using 10 microsatellite loci.

| Source | df | SS | MS | Est.Var | % |
| --- | --- | --- | --- | --- | --- |
| Among Pops | 13 | 310.595 | 22.185 | 0.342 | 9.5 |
| Among Indiv | 395 | 1385.840 | 3.508 | 0.251 | 7.0 |
| Within Indiv | 410 | 1233.000 | 3.007 | 3.007 | 83.5 |
| Total | 819 | 2929.435 |  | 3.600 | 100.0 |
