# Supplementary material for: Genetic and phenotypic differentiation of lumpfish (Cyclopterus lumpus) across the North Atlantic: implications for conservation and aquaculture
Source: PeerJ. 2018 Nov 20;6:e5974. doi: 10.7717/peerj.5974 (PMC6251346; doi:10.7717/peerj.5974)
Supplement: Table S11 [file peerj-06-5974-s012.docx]

**Table S11**. Effective number of migrants (*N*_m_) amongst 15 populations estimated using div-Migrate across 10 microsatellite loci, * denotes significant asymmetric gene flow at α=0.05.

|  | FB | CB | WB | Ha | Kl | VB | OH | We | Gu | Na | Av | Ro | KB | Öl | GS |
| --- | --- | --- | --- | --- | --- | --- | --- | --- | --- | --- | --- | --- | --- | --- | --- |
| FB |  | 0.639 | 0.465 | 0.066 | 0.083 | 0.102 | 0.068 | 0.057 | 0.053 | 0.076 | 0.076 | 0.050 | 0.096 | 0.047 | 0.053 |
| CB | 0.470 |  | 0.399 | 0.071 | 0.078 | 0.109 | 0.081 | 0.061 | 0.047 | 0.089 | 0.077 | 0.037 | 0.102 | 0.054 | 0.048 |
| WB | 0.247 | 0.325 |  | 0.073 | 0.099 | 0.099 | 0.084 | 0.072 | 0.059 | 0.091 | 0.095 | 0.043 | 0.086 | 0.046 | 0.054 |
| Ha | 0.076 | 0.089 | 0.094 |  | 0.192 | 0.246 | 0.270 | 0.161 | 0.144 | 0.158 | 0.104 | 0.141 | 0.241 | 0.108 | 0.134* |
| Kl | 0.078 | 0.070 | 0.077 | 0.181 |  | 0.441 | 0.276 | 0.190 | 0.204 | 0.197 | 0.227 | 0.134 | 0.232 | 0.070 | 0.076 |
| VB | 0.071 | 0.088 | 0.099 | 0.234 | 0.611 |  | 0.503 | 0.294 | 0.227 | 0.329 | 0.320 | 0.213 | 0.505 | 0.125 | 0.115* |
| OH | 0.075 | 0.119 | 0.082 | 0.231 | 0.376 | 0.548 |  | 0.351 | 0.497 | 0.574 | 0.212 | 0.238 | 0.345 | 0.130 | 0.119 |
| We | 0.069 | 0.086 | 0.094 | 0.226 | 0.297 | 0.359 | 0.514 |  | 1.000 | 0.303 | 0.194 | 0.197 | 0.200 | 0.132* | 0.138* |
| Gu | 0.062 | 0.080* | 0.088 | 0.149 | 0.232 | 0.259 | 0.509 | 0.825 |  | 0.290 | 0.205 | 0.166 | 0.157 | 0.113* | 0.108* |
| Na | 0.069 | 0.103 | 0.082 | 0.183 | 0.473 | 0.546 | 0.892 | 0.378 | 0.365 |  | 0.349 | 0.302 | 0.479 | 0.100 | 0.091 |
| Av | 0.082 | 0.120 | 0.101 | 0.119 | 0.331 | 0.347 | 0.242 | 0.242 | 0.185 | 0.323 |  | 0.115 | 0.209 | 0.073 | 0.066 |
| Ro | 0.071 | 0.046 | 0.090* | 0.216 | 0.507* | 0.306 | 0.284 | 0.178 | 0.205 | 0.181 | 0.143 |  | 0.259 | 0.062 | 0.078 |
| KB | 0.078 | 0.117 | 0.106 | 0.207 | 0.355 | 0.636 | 0.457 | 0.272 | 0.189 | 0.337 | 0.232 | 0.218 |  | 0.127 | 0.110 |
| Öl | 0.056 | 0.084 | 0.046 | 0.055 | 0.057 | 0.058 | 0.093 | 0.062 | 0.056 | 0.076 | 0.090 | 0.053 | 0.137 |  | 0.441 |
| GS | 0.057 | 0.064 | 0.039 | 0.053 | 0.055 | 0.057 | 0.085 | 0.049 | 0.048 | 0.081 | 0.093 | 0.046 | 0.141 | 0.245 |  |
