# Supplementary material for: Genetic and phenotypic differentiation of lumpfish (Cyclopterus lumpus) across the North Atlantic: implications for conservation and aquaculture
Source: PeerJ. 2018 Nov 20;6:e5974. doi: 10.7717/peerj.5974 (PMC6251346; doi:10.7717/peerj.5974)
Supplement: Table S12 [file peerj-06-5974-s013.docx]

**Table S12.** Effective population size (*N*_e_) and 95% confidence intervals estimated using the LD method in NeEstimator for populations containing at least 19 individuals, genotyped using 10 microsatellite loci.

|  | | NeEstimator | | | |
| --- | --- | --- | --- | --- | --- |
| Population | Country | | Estimated *N*_e_ | 95 CI | JackKnife Method |
| FB | USA | | 254.8 | 64.7 | 65.0 |
| CB | USA | | 75.4 | 38.9 | 36.0 |
| WB | Canada | | ∞ | 204.9 | 106.5 |
| Ha | Iceland | | 43.0 | 25.6 | 23.2 |
| Kl | Faroe Is. | | 30.3 | 21.1 | 19.9 |
| VB | Ireland | | 97.9 | 48.7 | 40.3 |
| OH | Scotland | | 205.6 | 66.0 | 60.9 |
| We∞ | England | | ∞ | ∞ | 536.1 |
| Gu | England | | ∞ | 113.5 | 91.1 |
| Na | Norway | | 72.2 | 32.1 | 25.9 |
| Av | Norway | | 62.3 | 33.4 | 30.9 |
| Ro | Norway | | 19.2 | 10.7 | 10.6 |
| KB | Denmark | | 70,147.8 | 84.5 | 79.6 |
| GS | Sweden | | 454.6 | 40.0 | 26.4 |

|  |  |  |  |  |  |  |  |  |  |  |  |  |  |
| --- | --- | --- | --- | --- | --- | --- | --- | --- | --- | --- | --- | --- | --- |
|  |  |  |  |  |  |  |  |  |  |  |  |  |  |
|  |  |  |  |  |  |  |  |  |  |  |  |  |  |
