# Supplementary material for: Genetic and phenotypic differentiation of lumpfish (Cyclopterus lumpus) across the North Atlantic: implications for conservation and aquaculture
Source: PeerJ. 2018 Nov 20;6:e5974. doi: 10.7717/peerj.5974 (PMC6251346; doi:10.7717/peerj.5974)
Supplement: Table S13 [file peerj-06-5974-s014.docx]

**Table S13**. Wilcoxon signed-rank tests for heterozygosity excess and heterozygosity deficiency across 10 microsatellite loci in 15 populations calculated using Bottleneck, under the Two-Phase Model of Mutation (TPM) and the Stepwise Mutation Model (SMM), * denotes significant value after Bonferroni correction (*P* = 0.0033).

| Population |  | TPM | |  | SSM | |
| --- | --- | --- | --- | --- | --- | --- |
|  | Country | Het excess | Het deficiency |  | Het excess | Het deficiency |
| FB | USA | 0.754 | 0.278 |  | 0.991 | 0.012 |
| CB | USA | 0.188 | 0.839 |  | 0.958 | 0.053 |
| WB | Canada | 0.500 | 0.539 |  | 0.984 | 0.042 |
| Ha | Iceland | 0.784 | 0.688 |  | 0.784 | 0.246 |
| Kl | Faroe Is. | 0.461 | 0.577 |  | 0.991 | 0.012 |
| VB | Ireland | 0.884 | 0.138 |  | 0.999 | 0.002* |
| OH | Scotland | 0.754 | 0.278 |  | 0.998 | 0.003* |
| We | England | 0.754 | 0.278 |  | 0.947 | 0.065 |
| Gu | England | 0.423 | 0.615 |  | 0.984 | 0.042 |
| Na | Norway | 0.920 | 0.910 |  | 0.988 | 0.016 |
| Av | Norway | 0.500 | 0.539 |  | 0.984 | 0.042 |
| Ro | Norway | 0.500 | 0.539 |  | 0.138 | 0.884 |
| KB | Denmark | 0.754 | 0.278 |  | 0.947 | 0.065 |
| Öl | Sweden | 0.754 | 0.278 |  | 0.884 | 0.138 |
| GS | Sweden | 0.500 | 0.539 |  | 0.839 | 0.188 |
