# Supplementary material for: Genetic and phenotypic differentiation of lumpfish (Cyclopterus lumpus) across the North Atlantic: implications for conservation and aquaculture
Source: PeerJ. 2018 Nov 20;6:e5974. doi: 10.7717/peerj.5974 (PMC6251346; doi:10.7717/peerj.5974)

Figure S1. Likelihood plots showing number of clusters present in STRUCTURE analysis of 15 populations genotyped using 10 microsatellite loci, informed by median of means (MedMeaK), maximum of means (MaxMeaK), median of medians (MedMeaK) and maximum of medians (MaxMedK) implemented in STRUCTURESELECTOR.

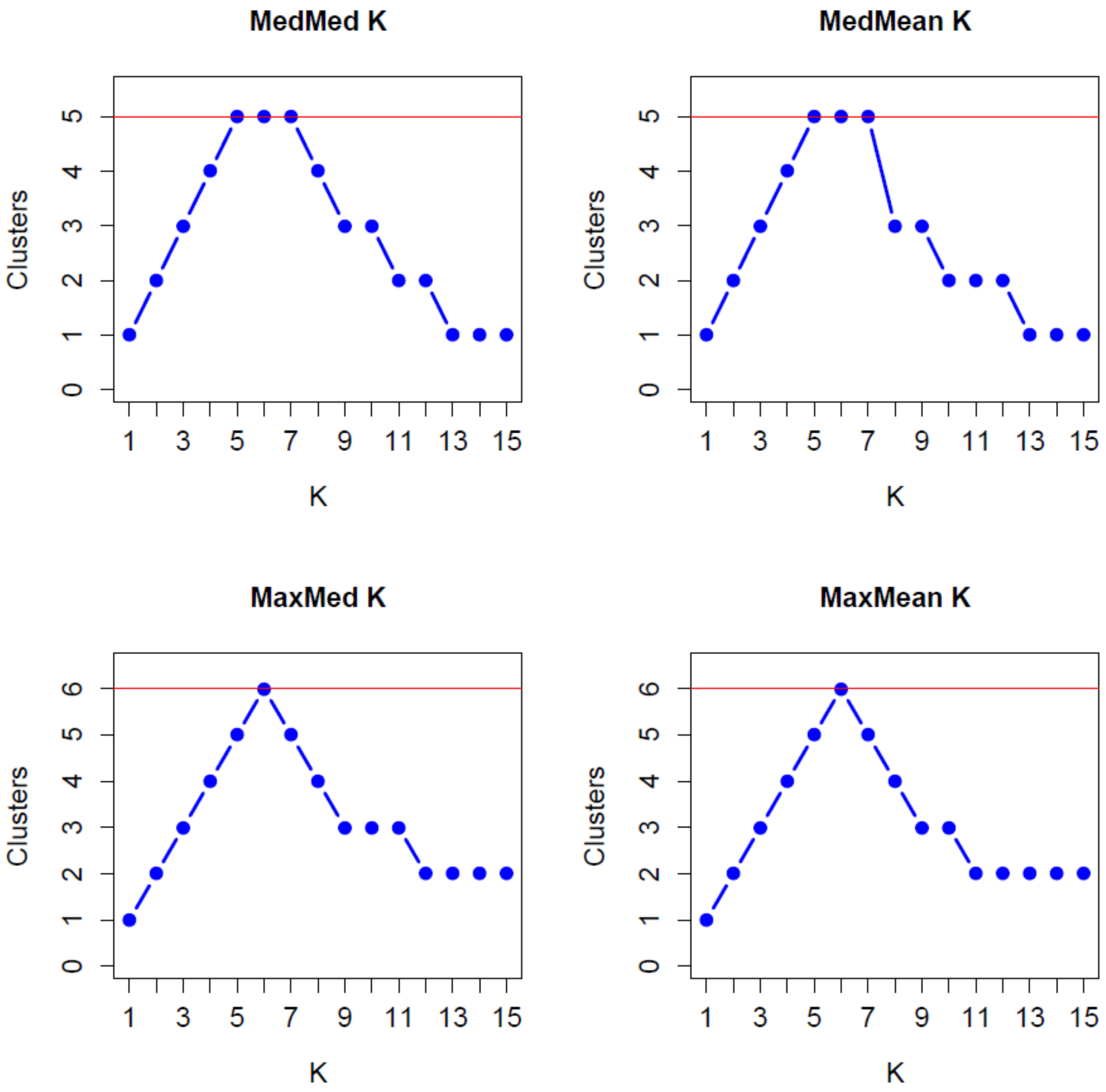

Supplement: Figure S1 [file peerj-06-5974-s015.pdf]
