# Supplementary material for: Genetic and phenotypic differentiation of lumpfish (Cyclopterus lumpus) across the North Atlantic: implications for conservation and aquaculture
Source: PeerJ. 2018 Nov 20;6:e5974. doi: 10.7717/peerj.5974 (PMC6251346; doi:10.7717/peerj.5974)

Figure S2. Likelihood plot showing mean Deviance Information Criterion (DIC) of the lowest 10 DIC values per K_{MAX} within lowest 10% of DIC, implemented in TESS analysis using 15 populations genotyped with 10 microsatellite loci.

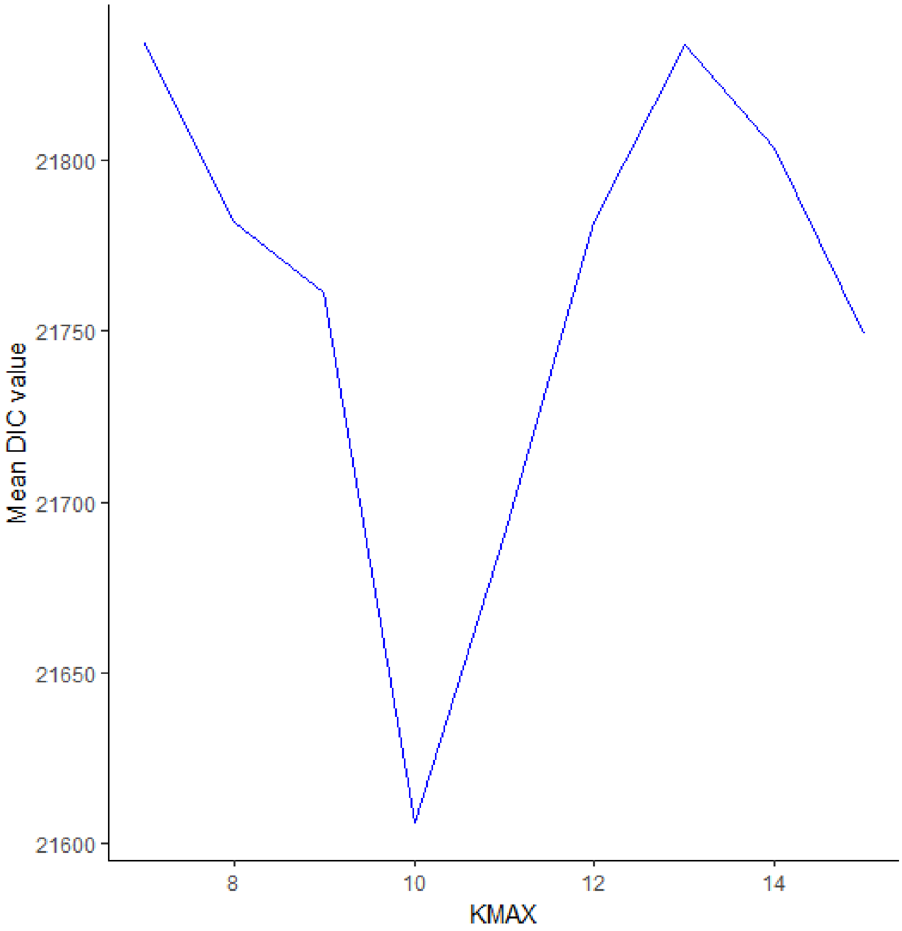

Supplement: Figure S2 [file peerj-06-5974-s016.pdf]
